# Delayed Extubation of a Patient With Multiple Sclerosis

**DOI:** 10.7759/cureus.49409

**Published:** 2023-11-25

**Authors:** Alexander Duong, Tomohiro Yamamoto, Satoshi Yamamoto

**Affiliations:** 1 Department of Anesthesiology, University of Texas Medical Branch, Galveston, USA; 2 Department of Medicine, Gunma University School of Medicine, Maebashi, JPN

**Keywords:** multiple sclerosis, delayed emergence, delayed extubation, gastroparesis, general anesthesiology

## Abstract

The potential complications associated with gastroparesis in the perioperative setting for patients with multiple sclerosis (MS) are inadequately recognized. While gastroparesis is commonly associated with diabetes mellitus-induced neuropathy and postsurgical complications, its prevalence and impact on patients with MS are less understood. This is particularly crucial as the systemic autoimmune nature of MS may extend its neurological effects to the gastrointestinal (GI) tract. In this context, we present a case wherein undiagnosed gastroparesis significantly contributed to postoperative challenges, leading to delayed extubation in a patient with MS. This underscores the importance of considering gastroparesis as a potential differential diagnosis and developing a comprehensive approach to evaluating and managing MS patients, which may help mitigate perioperative complications and inform tailored anesthetic management strategies.

## Introduction

Gastroparesis is the presence of delayed gastric emptying without mechanical obstruction. Commonly reported upper gastrointestinal (GI) tract symptoms include nausea and vomiting, followed by abdominal distension, pain, postprandial fullness, and early satiety [1,2]. The prevalence of gastroparesis is associated with a number of conditions. The majority of documented cases are attributed to diabetes mellitus-induced neuropathy, postsurgical complications, and idiopathic etiologies [1]. However, gastroparesis complicating multiple sclerosis (MS) is less known and may be a more frequent occurrence than previously recognized [3]. There is a report that found 16% of patients with MS had delayed gastric emptying [4]. We describe a challenging case of gastroparesis leading to a delayed extubation for a patient with MS.

## Case presentation

A 56-year-old male, weighing 95 kg (BMI 25), was scheduled for suprapubic catheter with cystoscopy. His past medical history was significant for MS, paraplegia, gastroesophageal reflux disease, hypertension, neurogenic bladder, and recurrent urinary tract infection secondary to chronic Foley use.

General anesthesia was induced with propofol, fentanyl, and rocuronium (1.2 mg/kg). The patient was intubated with an endotracheal tube using direct laryngoscopy with a Mac 3 blade (IntuBrite, Salter Labs, Arvin, California, United States). Rapid sequence intubation was chosen due to the patient's past medical history of controlled acid reflux disease. The patient did not report having any common symptoms of gastroparesis and reflux disease. Maintenance was done with sevoflurane under volume-controlled mode of ventilation. After the surgery was completed, the Train of Four (TOF) was 3/4 and confirmed 4/4 after reversal with sugammadex (2 mg/kg). Post emergence, the patient was awake and following verbal commands. However, he was not extubated due to high end-tidal carbon dioxide (ETCO2), erratic capnometry pattern, and low tidal volumes. Decision was made to transfer patient to post-anesthesia care unit (PACU) for further management.

**Figure 1:**
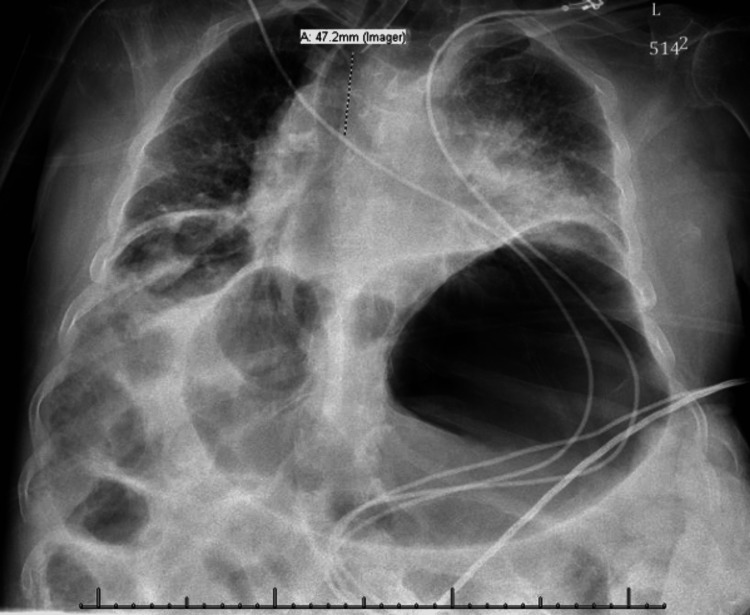
Chest X-ray showing large gastric bubble pre-OG tube placement OG: orogastric

**Figure 2:**
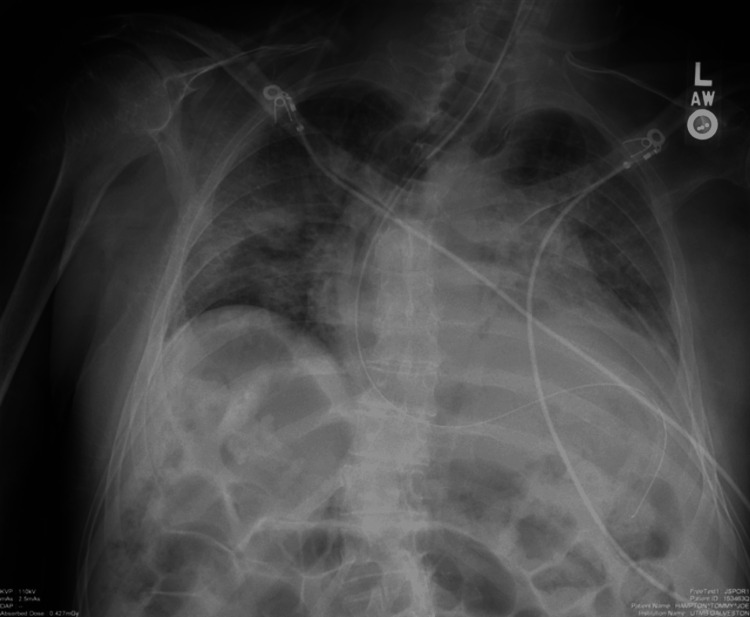
Chest X-ray after OG tube placement OG: orogastric

In PACU, the immediate postoperative chest X-ray revealed that the stomach was markedly distended with air with mild dilation of bowel loops in the upper abdomen. Findings also demonstrated very low lung volumes bilaterally (Figure 1). An orogastric (OG) tube was placed that allowed for the decompression of a large amount of air from the stomach. A repeat X-ray done after OG tube insertion confirmed the resolution of the gastric bubble (Figure 2). The patient was subsequently extubated and able to breathe on his own without further respiratory weakness.

During the postoperative interview, the patient revealed it was very common for him to have a large amount of air trapped in his stomach and that he typically has difficulty burping, indicating a high likelihood that the patient had a chronic condition. Based on the postoperative X-ray, it would be appropriate to conclude that this patient had mild compression atelectasis, presumably due to the increase in intrathoracic pressure created by the presence of the large gastric bubble.

## Discussion

MS is a chronic demyelinating disorder of the brain and spinal cord, characterized by remissions and relapses of variable neurological function. The prevalence of MS ranges from 250,000 to 350,000 patients in the United States alone, with a higher prevalence among women than men [5]. This inflammatory response eventually damages the blood-brain barrier, resulting in areas of multi-focal demyelination and glial scarring and leading to permanent neurological deficits and disability [6]. Because clinical symptoms vary depending on the location of the lesion in the central nervous system (CNS), neurological dysfunction may be affected in diverse organ systems, including within the GI viscera [7].

Commonly recognized manifestations of MS include visual impairment, sensory deficits, musculoskeletal weakness, and autonomic dysfunction; however, as indicated by this patient's case, upper GI manifestations should also be considered in the pleomorphic clinical presentation of this systemic autoimmune disease [6,7]. Though not frequently diagnosed, gastroparesis as a consequence of MS has been discussed in some case reports [8]. In 1981, Graves reported on a patient in which the finding of complete gastric outlet obstruction was the sole presenting symptom of MS [9]. In 1984, Gupta described a patient with a 14-year history of MS who developed gastroparesis. Gupta attributed his GI involvement to visceral CNS neuropathy [3]. In 2006, Raghav et al. reported on a case of disabling gastroparesis and dysphagia seen in a patient with a previous history of MS [8].

Gastric motility is mediated by a complex interplay of neural and humoral mechanisms [8]. An intact vagal nerve is essential for the coordination of gastric contractile activity within the parasympathetic nervous system. The enteric nervous system can also independently regulate motility by way of initiating migrating motor complexes (MMC) that expel undigested food from the stomach to the duodenum during the fasting state. Demyelination of the vagal nerve nuclei in the presence of a functioning inhibitory sympathetic input could contribute to the delay of proper gastric emptying [10]. There is also a study that suggests the pathophysiology between MS and gastroparesis may be due to areas of increased signal intensity within the medulla. These changes may have affected the parasympathetic fibers that stimulate gastric motility, ultimately leading to gastroparesis [11].

The neurotransmitter nitric oxide (NO) regulates the non-adrenergic, non-cholinergic smooth muscle relaxation governed by the myenteric plexus of the intestinal tract [12]. Reduced availability of NO may impair the gastric distension-induced accommodation reflex and relaxation of the pyloric sphincter [13]. Medications used to treat MS or its sequelae may have GI implications. Therapeutic management consists of multiple agents directed towards improving symptoms and preventing the further progression of disease. During acute attacks, corticosteroids are the mainstay of symptomatic treatment [5,10]. In studies performed in animal models, glucocorticoids have been shown to induce gastroparesis through the depletion of L-arginine in stomach cells, impeding the production of NO required for normal motor gastric function [14]. However, gastroparesis is still thought to be an unusual complication of MS, with disease onset occurring after many years [10].

The pathogenesis of gastroparesis as it relates to our patient suggests that he may have had asymptomatic chronic gastroparesis due to MS, which could have been exacerbated following anesthesia or surgery. [5]. In this case, he had an established history of MS and had already been displaying autonomic features such as neurogenic bladder. While our patient had not been previously diagnosed with gastroparesis, he had been experiencing frequent episodes of belching associated with excess swallowed air. Perioperative stress may have further reduced his gastric emptying; however, prompt clinical recognition of his deteriorating condition enabled immediate OG tube insertion and successful decompression of his stomach.

## Conclusions

Gastroparesis as a consequence of MS is poorly understood and poorly described in literature. Awareness of gastroparesis as a postoperative risk is imperative in the anesthetic management of the population affected by MS. Recognizing gastrointestinal motility deficits is a crucial component of evaluating symptomatic patients in need of medical intervention. If indicated, we recommend utilizing imaging modalities, such as chest X-ray or bedside ultrasound, to assess a patient's postoperative functional status. Furthermore, OG tube placement may be considered for air decompression before immediate extubation in patients with MS. Lastly, irrespective of patient history, extubation should not be performed when ETCO2 is unreasonably high or when obvious cardiopulmonary dysfunction exists. Our patient's clinical course highlights the importance of incorporating gastroparesis on the differential when there is suspicion of respiratory distress in a patient with MS.
